# Predictive model for preoperative deep vein thrombosis in distal femur fractures using inflammatory blood markers: a single-center retrospective cohort study

**DOI:** 10.7717/peerj.21531

**Published:** 2026-07-23

**Authors:** Xin Deng, Junfeng Wan, Xin Huang, Yang Wen, Jiaping Lan, Lei Li, Yang Liu

**Affiliations:** Suining Central Hospital, Suining, China

**Keywords:** Distal femoral fracture, Deep vein thrombosis, Risk factor, Nomogram, Blood index

## Abstract

**Background:**

The incidence of distal femur fractures (DFF) has shown a continuous annual increase. Preoperative deep vein thrombosis (DVT) of the lower limbs in DFF patients is associated with unfavorable clinical outcomes. Inflammation has been recognized as an essential contributor to thrombus formation, drawing increasing research attention in recent years.

**Objectives:**

This study aimed to construct a predictive model utilizing inflammatory blood indicators to estimate the preoperative risk of DVT in patients with DFF.

**Methods:**

A retrospective analysis was performed on 493 eligible patients treated for DFF, admitted to a single-center, between January 2022 and September 2025. Clinical baseline characteristics and laboratory parameters were collected. Independent risk factors for preoperative lower limb DVT were identified through univariate and multivariate binary logistic regression analyses. The predictive model was established using R 4.2.0, with patients randomly allocated to training and validation cohorts in a 7:3 ratio. Receiver operating characteristic (ROC) curves and corresponding areas under the curve (AUC) were computed for internal validation. Model performance was further assessed using calibration curves (CC) and decision curve analysis (DCA) in both cohorts.

**Results:**

A total of 493 eligible patients were analyzed, comprising 205 cases with DVT and 288 without DVT. Univariate and multivariate binary logistic regression identified neutrophil- to-lymphocyte (NLR) (OR = 0.86, 95% confidence interval (CI) [0.774–0.95], *P* = 0.004), lymphocyte-to-monocyte ratio (LMR) (OR = 1.545, 95% CI [1.3–1.851], *P* = 0.000), white blood cell count (OR = 1.245, 95% CI [1.152–1.353], *P* = 0.000), neutrophil count (OR = 1.563, 95% CI [1.398–1.766], *P* = 0.000), lymphocyte count (OR = 0.151, 95% CI [0.077–0.285], *P* = 0.000), and eosinophil count (OR = 2.343, 95% CI [0.657–8.447], *P* = 0.19) as independent variables associated with preoperative lower limb DVT in DFF patients. Incorporating age with these indicators, a predictive model was developed. The ROC analysis demonstrated AUC values of 0.926 and 0.939 in the training and validation cohorts, respectively.

**Conclusions:**

Among the preoperative inflammatory indicators evaluated, NLR, LMR, white blood cell count, neutrophil count, lymphocyte count, and eosinophil count were identified as independent determinants of preoperative lower limb DVT in patients with DFF. The predictive model constructed based on independent risk factors has excellent predictive efficacy and clinical application value.

## Background

Venous thromboembolism (VTE) comprises DVT and pulmonary thromboembolism (PTE). Among these, DVT represents a frequent and severe perioperative complication in patients with distal femur fractures (DFF). DFF accounts for approximately 4–6% of all femoral fractures and is predominantly characterized by comminution and instability (Claireaux et al., 2022). The knee region contains an extensive venous network, and the traction exerted by the gastrocnemius muscle often results in posterior displacement of fracture fragments in DFF. These displaced fragments may damage adjacent vessels, particularly the femoral and popliteal veins (Seddio et al., 2025), thereby markedly increasing the likelihood of DVT. In patients with DFF, preoperative lower limb DVT not only delays surgical intervention and disrupts the optimal timing for fixation, adversely influencing postoperative outcomes, but also poses a severe intraoperative hazard. Undiagnosed DVT prior to surgery may lead to thrombus dislodgement, allowing emboli to migrate through the bloodstream into the pulmonary artery, resulting in pulmonary embolism—an acute, potentially fatal event (Lapidus et al., 2007). Furthermore, DVT formation may precipitate post-thrombotic syndrome, manifested as persistent leg swelling, pain, skin pigmentation, and ulceration (Li et al., 2020), which substantially diminishes patient quality of life while increasing both physical distress and financial burden.

Developing a risk prediction model based on patients’ general conditions and hematological parameters at admission may offer a practical solution to this challenge. Although multiple clinical scales for DVT prediction have been established, their accuracy and consistency remain controversial. The Wells score, for example, incorporates comprehensive variables for DVT risk assessment but suffers from subjective components and limited specificity, reducing its applicability in acute trauma cases (Silveira et al., 2015). Similarly, the Caprini (2005) risk assessment model, while objective and inclusive of over 30 variables across diverse dimensions, involves a cumbersome procedure unsuitable for rapid clinical decision-making. Furthermore, existing models lack specificity for trauma populations, particularly for varying fracture types characterized by location, displacement, comminution, and extent of involvement. Recently, research attention has increasingly shifted toward the role of inflammation in thrombogenesis. Inflammatory biomarkers and their derived ratios obtained from blood tests—key indicators of systemic inflammatory status—appear closely linked to the development of preoperative DVT in patients undergoing major abdominal surgery (DFF). Ratios such as neutrophil-to-lymphocyte (NLR) and platelet-to-lymphocyte (PLR), along with related indices, have demonstrated associations with thrombotic risk across diverse pathological conditions (Niu et al., 2022; Liu et al., 2020). Integrating these inflammatory indicators and derived ratios with additional clinical parameters in predictive modeling may enhance the accuracy of preoperative lower extremity DVT risk estimation in DFF patients, thereby providing more specific guidance for clinical prevention and management.

Given this, this study retrospectively analyzed the independent risk factors associated with preoperative lower extremity DVT in DFF patients by examining admission blood test indicators and their derived ratios. A nomogram prediction model was established to enhance the accuracy of preoperative DVT risk assessment in this population, thereby offering more reliable guidance for clinical decision-making.

## Methods

### Study subjects

The data for this study originated from the electronic medical record system of DFF patients diagnosed in Suining Central Hospital, Sichuan Province, China, between January 2022 and September 2025. This system comprehensively documented the entire diagnostic and therapeutic course from admission to discharge, including demographic information, physical examination findings, imaging results, and laboratory parameters (including blood routine tests, coagulation profiles, and other relevant indicators). During the data entry process, a two-person entry method was adopted. Two orthopedic physicians, who had received professional data entry training, were assigned to independently input the electronic medical record information of patients into the pre-designed electronic data form. Upon completion, specialized verification software compared both datasets variable by variable. Inconsistencies were rechecked against the original records, and the discrepancies were jointly reviewed and resolved by the data entry personnel to ensure data integrity and accuracy. This study was approved by Medical Research Ethics Committee of Suining Central Hospital (NO. KYLLKS20250213).

**Figure 1:**
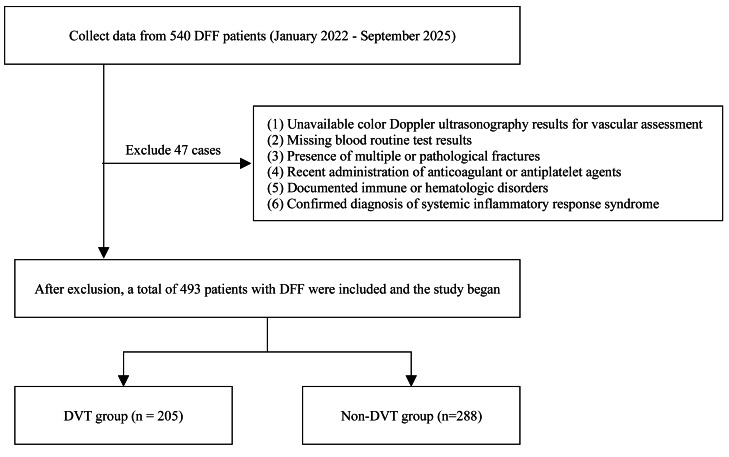
Flowchart.

Inclusion criteria: (1) History of trauma involving the knee joint; (2) Radiographic and CT evidence indicating cortical discontinuity or marked displacement of the distal femur on the affected side; (3) Availability of complete preoperative laboratory data, including white blood cell, neutrophil, lymphocyte, and platelet counts, prothrombin time, activated partial thromboplastin time, and fibrinogen levels, allowing precise calculation of inflammatory indices and derived ratios as well as evaluation of coagulation profiles; (4) Patients who underwent surgical treatment after admission; (5) Absence of any specific therapeutic intervention at the time of admission. Exclusion criteria: (1) Unavailable color Doppler ultrasonography results for vascular assessment; (2) Missing blood routine test results; (3) Presence of multiple or pathological fractures; (4) Those who had taken anticoagulant or antiplatelet drugs before the fracture; (5) Documented immune or hematologic disorders; (6) Confirmed diagnosis of systemic inflammatory response syndrome. The screening process of enrolled subjects is illustrated in Fig. 1.

### Data collection

Baseline clinical information and laboratory parameters, including complete blood count, serum biochemistry, and coagulation profiles, were collected. Clinical variables comprised sex, age, height, weight, body mass index (BMI), and medical histories of hypertension, diabetes, hepatitis, alcohol intake, and smoking. Hypertension and diabetes histories referred to prior medical diagnoses of the respective conditions. Ratios derived from inflammatory markers were computed based on preoperative hematological results: neutrophil-to-lymphocyte ratio (NLR=neutrophil count/lymphocyte count), platelet-to-lymphocyte ratio (PLR=platelet count/lymphocyte count), monocyte-to-lymphocyte ratio (MLR=monocyte count/lymphocyte count), neutrophil-to-eosinophil ratio (NAR=neutrophil count/eosinophil count), lymphocyte-to-monocyte ratio (LMR=lymphocyte count/monocyte count), monocyte-to-eosinophil ratio (MAR=monocyte count/eosinophil count), aspartate aminotransferase-to-lymphocyte ratio (ALRI), aspartate aminotransferase-to-neutrophil ratio (ANRI), aspartate aminotransferase-to-platelet ratio (APRI), and fibrinogen-to-albumin ratio (FAR).

The diagnosis of lower extremity deep vein thrombosis was established through color Doppler ultrasonography of the lower limb vessels. The diagnostic criteria, based on the standards proposed by Dauzat et al. (1997), include: (1) solid echogenic material of variable size within the venous lumen; (2) persistent venous filling; and (3) absence or reduction of blood flow during probe compression or movement across the thrombus.

Data collection followed a predefined protocol to ensure consistency and precision. Missing information was retrieved, when possible, by reviewing medical records or consulting the attending physicians. In cases where critical data remained incomplete, participant exclusion was determined according to the prespecified criteria.

### Statistical analysis

The dataset was classified into DVT and non-DVT groups according to the presence of preoperative lower extremity DVT in DFF patients. For continuous variables, normality was assessed *via* the Shapiro–Wilk test. Data conforming to a normal distribution were presented as mean ± standard deviation and analyzed using independent one-way ANOVA. Non-normally distributed data were expressed as quartiles (25th percentile, 75th percentile) and compared between groups using the Kruskal–Wallis test. Categorical variables were summarized as frequency (percentage) and analyzed with the chi-square test. A two-sided *P* value < 0.05 indicated statistical significance.

Secondly, the glm function was used for univariate binary logistic regression analysis (that is, each variable was sequentially added to the model. When the independent variable was a categorical variable, the minimum value group was used as the reference group; when it was a continuous variable, the continuous variable was directly included in the binary logistic regression model. To avoid missing relevant risk factors, the ROC curve was applied. The value at which the Youden index was the largest was selected as the cut-off value, and the continuous variable was replaced with a binary variable, followed by binary logistic regression analysis. In the results of the multivariate analysis, the variables with *P* < 0.05 were regarded as independent risk factors for the occurrence of lower extremity DVT in DFF patients before surgery.

The dataset of 493 DFF patients was randomly divided into a training cohort (*n* = 346) and a validation cohort (*n* = 147) at a 7:3 ratio using SPSS 26.0. Based on multivariate logistic regression outcomes, a predictive model integrating independent risk factors with age was developed in R 4.2.0. Model performance was assessed through receiver operating characteristic (ROC) analysis and corresponding area under the curve (AUC) values, while calibration plots were employed to estimate the average prediction error. Decision curve analysis (DCA) further evaluated the model’s predictive capability and clinical applicability. The AUC, ranging from 0.5 to 1.0, reflects model discrimination, with higher values indicating stronger predictive accuracy. The calibration curve measured the agreement between predicted and observed probabilities of lower limb DVT, with greater overlap between the prediction and ideal curves representing superior consistency. The DCA, constructed on threshold probability and net benefit, quantified the clinical utility and potential decision-making advantage of the model.

## Results

### Univariate analysis of preoperative lower extremity deep vein thrombosis formation in DFF patients

The univariate analysis revealed that age, NLR, LMR, white blood cell count, neutrophil count, lymphocyte count, eosinophil count, alkaline phosphatase, creatinine, history of hypertension, history of diabetes, and alcohol consumption were associated with an increased risk of preoperative lower extremity DVT in DFF patients (*P* < 0.001) (Table 1).

**Table 1:** Results of univariate analysis.

Variable	*P* value	OR [95% CI]
Gender	0.973	1.006 (0.701–1.443)
Age (years)	*P* < 0.001	1.065 (1.052–1.08)
NLR	*P* < 0.001	1.159 (1.109–1.215)
NAR	0.337	1 (1–1.001)
LMR	*P* < 0.001	1.416 (1.258–1.614)
MAR	0.219	1.005 (0.997–1.013)
FAR	0.248	0.086 (0.001–5.067)
APRI	0.775	1.064 (0.69–1.632)
ANRI	0.603	1.004 (0.99–1.018)
ALRI	0.992	1 (0.997–1.003)
WBC (×10^9^/L)	*P* < 0.001	1.233 (1.175–1.298)
RBC (×10^12^/L)	0.398	0.923 (0.766–1.111)
NEUT (×10^9^/L)	*P* < 0.001	1.357 (1.284–1.44)
LYMPH (×10^9^/L)	0.008	0.721 (0.566–0.915)
MONO (×10^9^/L)	0.402	1.143 (0.836–1.563)
EO (×10^9^/L)	0.001	3.691 (1.727–7.975)
HGB (g/L)	0.727	0.999 (0.993–1.005)
MCV (fL)	0.112	1.02 (0.995–1.046)
MCH (pg)	0.749	1.009 (0.953–1.069)
MCHC (g/L)	0.079	0.99 (0.978–1.001)
PLT (×10^9^/L)	0.274	1.001 (1–1.002)
PDW (fL)	0.321	1.02 (0.98–1.062)
MPV (fL)	0.477	1.03 (0.95–1.117)
PT (s)	0.269	1.054 (0.96–1.157)
PT% (%)	0.597	0.998 (0.99–1.006)
INR	0.118	1.48 (0.906–2.424)
APTT (s)	0.36	1.012 (0.986–1.039)
FIB (g/L)	0.821	0.989 (0.896–1.091)
TT (s)	0.081	1.03 (0.996–1.066)
ALT (U/L)	0.072	1.002 (1–1.004)
ALP (U/L)	0.007	1.003 (1.001–1.005)
ALB (g/L)	0.087	1.001 (1–1.003)
TBIL (μmol/L)	0.219	1.01 (0.994–1.025)
UREA (μmol/L)	0.112	1.065 (0.986–1.151)
CREA (μmol/L)	*P* < 0.001	1.023 (1.012–1.034)
Height (cm)	0.321	1.008 (0.992–1.024)
Weight (kg)	0.407	0.995 (0.984–1.007)
Hypertension	0.027	1.501 (1.048–2.155)
Diabetes mellitus	0.004	1.71 (1.193–2.459)
History of hepatitis	0.388	0.853 (0.594–1.225)
History of alcohol use	0.005	1.685 (1.176–2.423)
Smoking history	0.09	1.365 (0.953–1.957)

**Notes.**

The annotations of each indicator in the table are the same as in the following tables.

DVT deep vein thrombosis NLR neutrophil-to-lymphocyte ratio NAR neutrophil-to-eosinophil ratio LMR lymphocyte-to-monocyte ratio MAR monocyte-to-eosinophil ratio FAR fibrinogen-to-albumin ratio APRI aspartate aminotransferase-to-platelet ratio ANRI aspartate aminotransferase-to-neutrophil ratio ALRI aspartate aminotransferase-to-lymphocyte ratio WBC white blood cell count RBC red blood cell count NEUT neutrophil count LYMPH lymphocyte count MONO monocyte count EO eosinophil count HGB hemoglobin MCV mean corpuscular volume MCH mean corpuscular hemoglobin MCHC mean corpuscular hemoglobin concentration PLT platelet count PDW platelet distribution width MPV mean platelet volume PT prothrombin time PT% prothrombin activity INR international normalized ratio APTT activated partial thromboplastin time FIB fibrinogen TT thrombin time ALT alanine transaminase ALP alkaline phosphatase ALB albumin TBIL total bilirubin UREA urea CREA creatinine

### Multivariate analysis of preoperative lower extremity deep vein thrombosis formation in DFF patients

Univariate analysis was first performed to identify potential risk factors, which were subsequently incorporated into a multivariate binary logistic regression model. Independent predictors were determined using a stepwise selection approach. The analysis indicated that NLR, LMR, white blood cell count, neutrophil count, lymphocyte count, and eosinophil count served as significant risk factors for the development of preoperative lower extremity DVT in DFF patients (*P* < 0.05) (Table 2).

**Table 2:** Results of multivariate analysis.

Variable	*P* value	OR [95% CI]
Age (years)	*P* < 0.001	1.078 (1.058–1.101)
NLR	0.004	0.86 (0.774–0.95)
LMR	*P* < 0.001	1.545 (1.3–1.851)
WBC (×10^9^/L)	*P* < 0.001	1.245 (1.152–1.353)
NEUT (×10^9^/L)	*P* < 0.001	1.563 (1.398–1.766)
LYMPH (×10^9^/L)	*P* < 0.001	0.151 (0.077–0.285)
EO (×10^9^/L)	0.19	2.343 (0.657–8.447)
ALP (U/L)	0.637	1.001 (0.998–1.004)
CREA (μmol/L)	0.07	1.015 (0.999–1.032)
Hypertension	0.484	1.23 (0.687–2.204)
Diabetes mellitus	0.25	1.397 (0.79–2.479)
History of alcohol use	0.377	1.294 (0.73–2.302)

### The construction of predictive models

**Figure 2:**
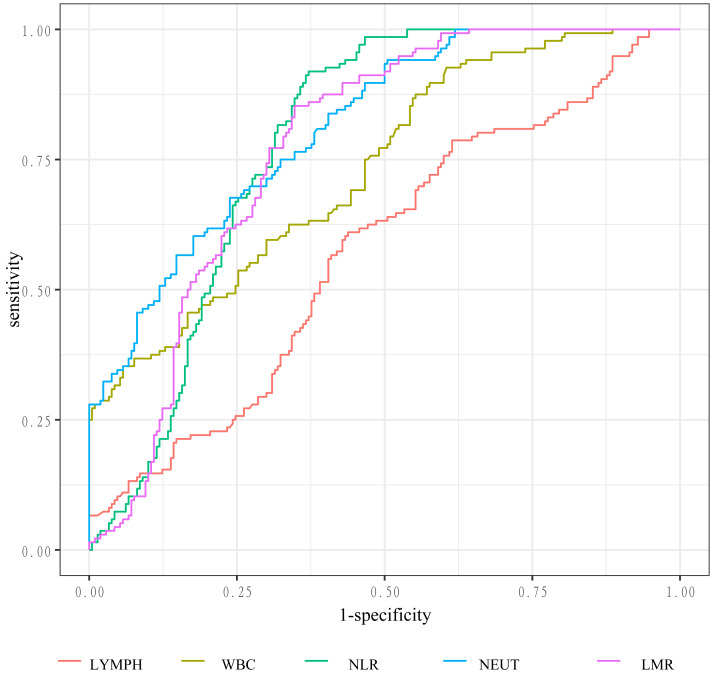
ROC curves of individual risk factors for predicting DVT independently.

**Figure 3:**
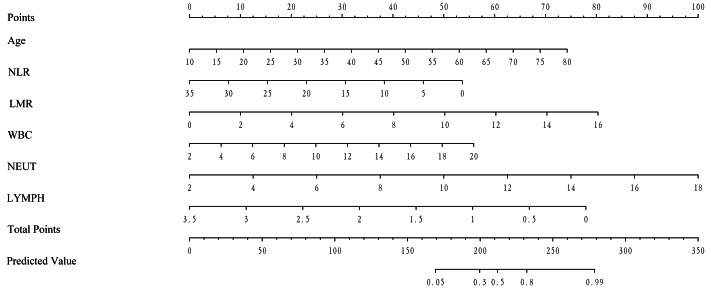
Nomogram model. This model assigns corresponding scores to the six values of Age, NLR, LMR, WBC, NEUT, and LYMPH, and determines the final risk of poor prognosis by matching the total score with the predict.

Based on Multivariate Logistic regression analysis, ROC curves for each variable were generated using R4.2.0 (Fig. 2). In the predictive model, each independent factor is represented by an individual scale axis. For instance, the age axis is segmented according to the observed value range, where higher age values correspond to higher scores. Similar axes are assigned to white blood cell count, absolute neutrophil count, absolute lymphocyte count, and PLR, with scale intervals determined by their respective regression coefficients and statistical relevance. The cumulative score is calculated by locating each factor’s value on its axis, projecting vertically to the total score line, and summing the results. The probability of preoperative DVT occurrence is then estimated by aligning the total score with the corresponding probability on the outcome scale (Fig. 3).

### Evaluation of the performance of predictive models

**Figure 4:**
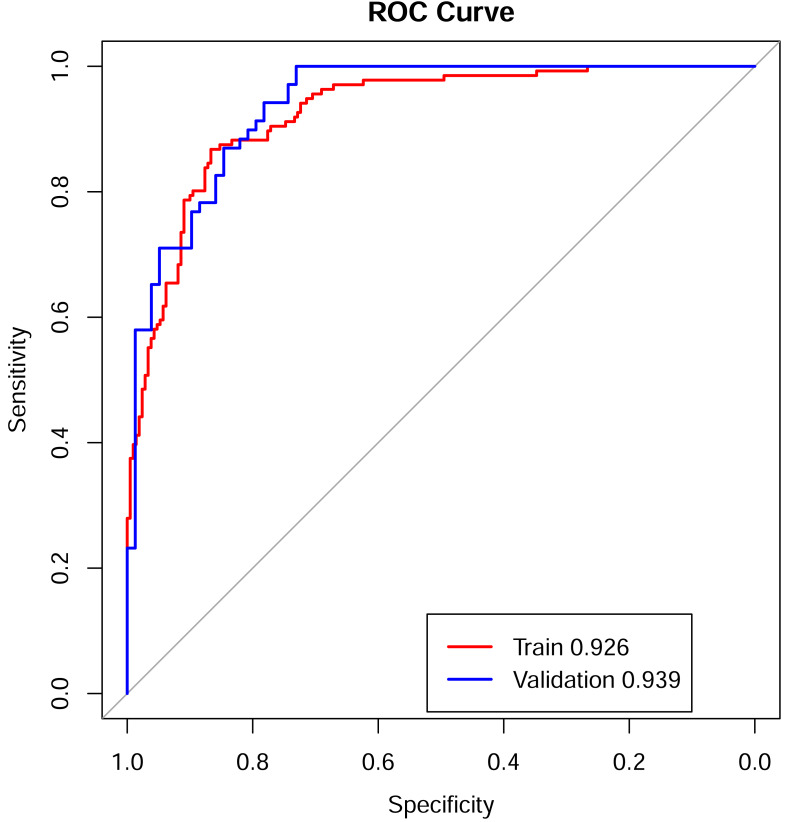
ROC curve of the model for predicting DVT.

**Figure 5:**
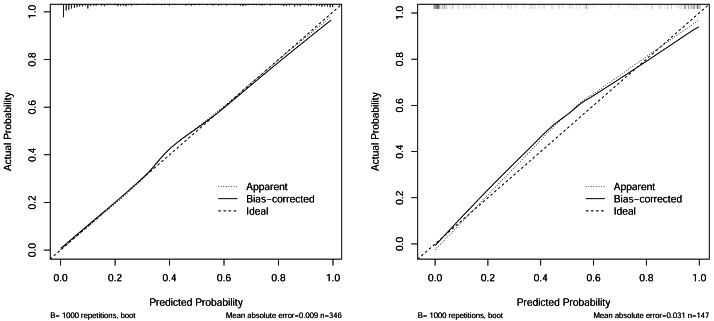
Calibration curve of the model for predicting DVT. After 1,000 bootstrap resamplings, the Brier score of the calibration curve for the training set was 0.1129 (95% CI [0.0906–0.1354]), and that for the validation set was 0.1063 (95% CI [0.0719–0.146]).

**Figure 6:**
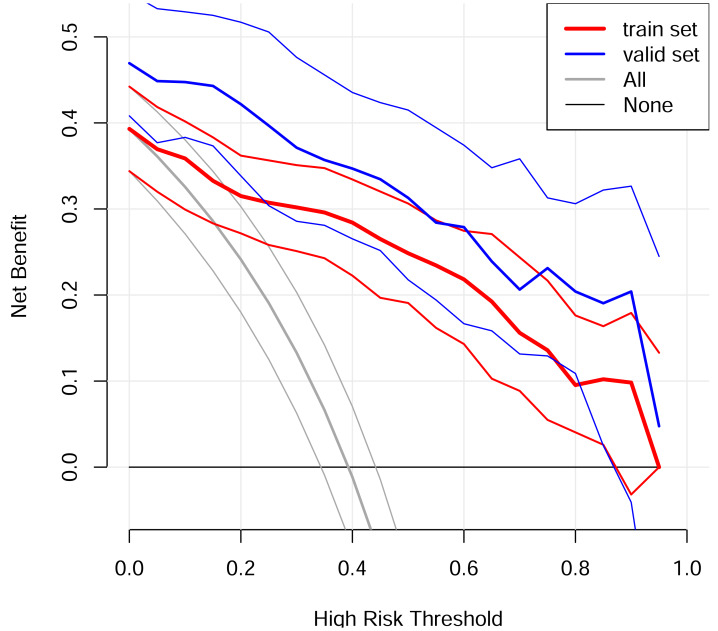
Decision curve of the model for predicting DVT.

In this study, 493 patients with DFF were randomly assigned in a 7:3 ratio to a training cohort (*n* = 346) and a validation cohort (*n* = 147). Baseline characteristics between the two cohorts were compared, revealing no statistically significant differences in data distribution except for the variable of hepatitis history (*P* > 0.05), confirming the success of randomization (Table 3). A nomogram model was established based on independent risk factors identified from the training cohort, and its predictive capacity was evaluated using ROC analysis. The AUC of the training cohort reached 0.926 (Fig. 4), reflecting strong discrimination. The calibration curve demonstrated an average prediction error of 0.009 (Fig. 5). DCA indicated substantial clinical utility across the threshold range of 0.01–0.95 (Fig. 6). Validation using the independent cohort yielded an AUC of 0.939 (Fig. 4), with a mean calibration error of 0.031 (Fig. 5). DCA further confirmed consistent clinical benefit within the same threshold interval (Fig. 6). Collectively, the prediction model derived from multivariate regression incorporating age exhibited high predictive accuracy and robust calibration in both the training and validation cohorts, demonstrating minimal deviation between predicted and observed outcomes.

**Table 3:** Baseline data of the training group and the validation group.

Variable	Training group	Validation group	*P* value
Gender Woman	192 (55%)	85 (58%)	0.705
Gender Man	154 (45%)	62 (42%)	–
Age (years)	46 (29, 59)	48 (31, 63)	0.227
NLR	5.253 (2.895, 8.501)	5.762 (3.203, 8.991)	0.156
NAR	29.259 (17.73, 74.02)	28.743 (17.807, 68.637)	0.894
LMR	1.796 (1.231, 2.993)	1.747 (1.281, 2.917)	0.903
MAR	3.851 (1.999, 8.391)	3.418 (2.025, 8.671)	0.665
FAR	0.023 (0.013, 0.058)	0.022 (0.013, 0.056)	0.872
APRI	0.262 (0.146, 0.479)	0.23 (0.128, 0.449)	0.586
ANRI	9.349 (4.827, 16.899)	8.994 (4.114, 14.204)	0.25
ALRI	50.058 (26.196, 84.795)	47.024 (26.181, 75.981)	0.622
WBC (×10^9^/L)	10.079 (6.872, 14.329)	9.461 (6.995, 13.032)	0.283
RBC (×10^12^ /L)	3.567 (2.706, 4.478)	3.359 (2.75, 4.008)	0.248
NEUT (×10^9^/L)	8.206 (5.168, 12.388)	9.253 (5.374, 13.571)	0.056
LYMPH (×10^9^/L)	1.761 (1.187, 2.427)	1.76 (1.124, 2.494)	0.958
MONO (×10^9^/L)	0.96 (0.546, 1.51)	0.942 (0.586, 1.607)	0.494
EO (×10^9^/L)	0.289 (0.105, 0.528)	0.315 (0.125, 0.547)	0.426
HGB (g/L)	101.588 (77.003, 133.184)	109.418 (77.166, 136.896)	0.294
MCV (fL)	92.197 (87.593, 98.425)	91.788 (86.331, 96.881)	0.106
MCH (pg)	29.623 (26.907, 32.123)	29.737 (26.63, 31.742)	0.964
MCHC (g/L)	326.081 (311.985, 336.516)	325.142 (310.323, 336.039)	0.528
PLT (×10^9^/L)	308.637 (182.94, 497.719)	306.326 (196.666, 467.526)	0.98
PDW (fL)	15.925 (12.451, 19.732)	15.937 (12.318, 19.905)	0.951
MPV (fL)	11.809 (9.814, 13.801)	11.955 (10.367, 13.758)	0.31
PT (s)	11.963 (10.8, 13.738)	12.24 (10.85, 14.521)	0.187
PT% (%)	108.901 (93.285, 125.567)	109.233 (91.698, 121.835)	0.52
INR	1.327 (1.01, 1.713)	1.196 (0.99, 1.601)	0.083
APTT (s)	32.487 (27.13, 38.531)	31.976 (27.631, 37.99)	0.888
FIB (g/L)	3.928 (2.552, 5.432)	3.576 (2.553, 5.716)	0.9
TT (s)	13.363 (7.672, 16.7)	13.83 (7.691, 17.1)	0.561
ALT (U/L)	107.506 (36.88, 187.87)	105.272 (34.134, 181.613)	0.565
ALP (U/L)	145.38 (81.158, 247.143)	179.117 (85.057, 266.528)	0.164
ALB (g/L)	180.494 (65.508, 317.417)	164.284 (60.875, 318.088)	0.806
TBIL (μmol/L)	23.36 (13.747, 33.596)	20.389 (12.639, 31.193)	0.191
UREA (μmol/L)	6.593 (4.591, 8.364)	6.55 (4.645, 8.992)	0.438
CREA (μmol/L)	59.403 (47.841, 75.096)	63.497 (48.618, 77.31)	0.331
Height (cm)	161.379 (152.636, 171.019)	160 (151.289, 168.05)	0.18
Weight (kg)	65 (54.003, 78.024)	61.382 (50, 76.302)	0.156
Hypertension No	174 (50%)	83 (56%)	0.247
Hypertension Yes	172 (50%)	64 (44%)	–
Diabetes mellitus No	197 (57%)	84 (57%)	1
Diabetes mellitus Yes	149 (43%)	63 (43%)	–
History of hepatitis No	137 (40%)	73 (50%)	0.049
History of hepatitis Yes	209 (60%)	74 (50%)	–
History of alcohol use No	189 (55%)	77 (52%)	0.72
History of alcohol use Yes	157 (45%)	70 (48%)	–
Smoking history No	195 (56%)	80 (54%)	0.767
Smoking history Yes	151 (44%)	67 (46%)	–

## Discussion

### Risk factors associated with lower extremity DVT

In this study, the author constructed a nomogram prediction model using five predictive variables (NLR, LMR, WBC, NEUT, LYMPH) of DFF patients before surgery and combined with age. This model can effectively predict the risk probability of DVT in DFF patients before surgery, and has good predictive efficacy and clinical application value.

The development of DVT is strongly associated with Virchow’s triad, comprising venous stasis, endothelial injury, and a hypercoagulable state of the blood (Stone et al., 2017). Following DFF, the local inflammatory cascade surrounding the fracture site may intensify venous hypercoagulability and endothelial damage in the lower limbs, thereby elevating the preoperative risk of DVT. Among diagnostic modalities, venography remains the recognized “gold standard” for confirming DVT; however, its invasive nature limits its routine clinical application (Sato et al., 2021). Plasma D-dimer, a specific degradation product of cross-linked fibrin generated through plasmin-mediated fibrinolysis, serves as an indicator of coagulation and secondary fibrinolytic activation. It demonstrates high sensitivity in the detection of acute DVT (Yoshiiwa et al., 2011; Jiang et al., 2016; Jiang et al., 2015). A negative D-dimer test reliably excludes thrombosis, whereas a positive result reflects excessive fibrinolytic activity without confirming thrombus formation. Nonetheless, its predictive accuracy for DVT in isolated lower-limb fractures remains limited (Niikura et al., 2015). Currently, color Doppler ultrasound is the preferred noninvasive technique for DVT diagnosis (Zhu et al., 2014). Accordingly, this study employed lower-extremity vascular color Doppler ultrasound findings to establish the diagnosis of DVT.

Multiple factors contribute to the development of preoperative DVT, yet the statistically significant risk determinants remain inadequately defined. Although prior investigations have examined risk factors for post-fracture DVT, including age, fracture type, and surgical duration (Lv et al., 2024; Tan et al., 2021), no nomogram prediction model incorporating inflammatory markers from admission blood tests has been established to assess preoperative lower extremity DVT in patients with DFF. Previous studies by Chen et al. (2026) and Yang et al. (2026). have demonstrated a close correlation between inflammatory indicators and deep vein thrombosis in fracture patients, which further supports the theoretical rationale of this study. Existing predictive frameworks for post-fracture DVT risk lack comprehensive integration of inflammatory indices. Moreover, early diagnosis of DVT remains challenging due to the invasiveness of venography, the temporal delay inherent in Doppler ultrasonography, and the limited specificity of plasma D-dimer assays, all of which may postpone timely intervention. Increasing evidence indicates a close association between lower extremity DVT formation and inflammatory mediators as well as blood cell-derived components, rendering this interaction a growing focus of current research. Developing a nomogram model based on inflammatory biomarkers to estimate preoperative DVT risk in DFF patients thus holds substantial clinical relevance.

Multiple factors are recognized to elevate the risk of preoperative lower extremity DVT in fracture patients, particularly those associated with inflammatory activation and enhanced coagulation, such as advanced age and prolonged preoperative immobilization (Sartori et al., 2023). In this study, demographic characteristics (gender, age, height, weight, BMI), medical history (hypertension, diabetes, hepatitis, and other chronic conditions), personal habits (alcohol consumption and smoking), as well as hematological parameters and derived inflammatory indices at admission were collected from DFF patients. To determine the association between preoperative inflammatory biomarkers and the incidence of lower extremity DVT, univariate and multivariate logistic regression analyses were conducted to identify independent predictors of DVT occurrence in this population. Logistic regression analysis was selected for its computational efficiency, clarity of interpretation, and ability to control for confounding factors, thereby ensuring the methodological rigor and reliability of the findings (Sperandei, 2014). The results demonstrated that NLR (OR = 0.86, 95% CI [0.774–0.95], *P* = 0.004), LMR (OR = 1.545, 95% CI [1.3–1.851], *P* = 0.000), white blood cell count (OR = 1.245, 95% CI [1.152–1.353], *P* = 0.000), neutrophil count (OR = 1.563, 95% CI [1.398–1.766], *P* = 0.000), lymphocyte count (OR = 0.151, 95% CI [0.077–0.285], *P* = 0.000), and eosinophils (OR = 2.343, 95% CI [0.657–8.447], *P* = 0.19) were independent risk factors for preoperative lower extremity DVT in DFF patients (*P* < 0.05). These parameters are readily obtainable and easily calculated, offering clinicians a practical and efficient approach to early DVT identification and management. Their application may assist in reducing disability and mortality while improving overall patient outcomes.

Extensive evidence-based research has established age as an independent determinant of DVT risk (Zhang et al., 2022; Akrivou et al., 2022; Barco et al., 2019; Li et al., 2022). Epidemiological data indicate that approximately 60% of venous thromboembolic events occur in individuals over 65 years of age (Tritschler & Aujesky, 2017), while the incidence in those above 80 is five to eight times higher than in the general population (Johnson, Eleazer & Rondina, 2016; LaMori et al., 2015). This elevated risk is primarily associated with age-related vascular and hemodynamic alterations. Functional decline of vascular endothelial cells and fragmentation of elastic fibers within vessel walls increase vascular fragility (Ribeiro-Silva et al., 2021). Concurrently, venous valve degeneration contributes to reflux dysfunction, and diminished muscle pump activity in the lower limbs slows venous return compared with younger individuals (Engbers, Vlieg & Rosendaal, 2010). Moreover, elevated plasma fibrinogen levels (Manzoni et al., 2024) and enhanced platelet aggregation (Montenont, Rondina & Campbell, 2019) in the elderly further promote thrombogenesis, fulfilling Virchow’s triad. Based on multivariate analysis, a nomogram model integrating six independent risk factors with age was established to assess its predictive accuracy and clinical utility. This model enables rapid and reliable preoperative estimation of DVT risk in patients with DFF, thereby supporting more informed clinical decision-making.

Inflammatory indicators, including NLR, LMR, white blood cell count, absolute neutrophil count, absolute lymphocyte count, and absolute eosinophil count, exhibited a strong association with preoperative lower extremity DVT in DFF patients. This correlation refines the conceptual basis of Virchow’s triad in trauma-induced thrombosis and introduces an alternative framework for assessing DVT risk through the inflammatory–coagulative interface. Elevated white blood cell count significantly increased the likelihood of DVT in patients with DFF, consistent with observations from large-scale cohort studies, suggesting that leukocytosis serves as a predictive factor for thrombus formation. During thrombogenesis, endothelial activation promotes the release of pro-inflammatory cytokines, recruiting monocytes and neutrophils and consequently elevating their circulating levels. These immune cells express adhesion molecules that enhance platelet aggregation and adhesion, thereby heightening thrombotic susceptibility (Buonacera et al., 2022). During inflammatory activation, elevated neutrophil counts trigger a systemic stress response accompanied by a decline in lymphocyte numbers, disrupting the balance of the monocyte-to-lymphocyte ratio. Evidence indicates that DVT patients exhibit increased serum levels of neutrophils and monocytes, with a concurrent reduction in lymphocytes (Yao et al., 2024). Historically viewed as short-lived effector cells, neutrophils are now recognized as active participants in thrombogenesis. Following endothelial injury, they accumulate at the damaged site before platelet activation occurs. In DFF, extensive fractures and trauma provoke a pronounced inflammatory response, inducing massive neutrophil mobilization into circulation. Through the release of neutrophil extracellular traps (NETs), neutrophils activate coagulation factors and promote thrombus formation (Block, Rossaint & Zarbock, 2022). As primary mediators of inflammation, elevated neutrophil counts signify intensified inflammatory activity. Being the most abundant leukocytes and a key component of innate immunity, neutrophils promote coagulation mainly through NET formation and activation by pathogens or receptors such as complement, antibodies, and cytokines (Shi et al., 2021). The primary constituents of NETs are DNA, histones, and various enzymes, among which DNA serves as the central mediator linking inflammation to thrombosis (Rangaswamy et al., 2021). Purified DNA and RNA can bind to and activate endogenous proteins, collectively enhancing thrombin generation. Histones, another major component of NETs, act as a newly identified category of damage-associated molecular patterns (DAMPs). Once released extracellularly, they induce organ injury through toll-like receptor (TLR)-dependent signaling or direct cytotoxicity toward epithelial and endothelial cells, thereby increasing thrombotic susceptibility (Block, Rossaint & Zarbock, 2022; Baroni Pietto et al., 2024). Lymphocytes are essential for immune homeostasis, and their reduction indicates dysregulation between inflammatory and immune responses, which may contribute to thrombogenesis through multiple mechanisms. Eosinophils have been characterized as indicators of a pre-thrombotic state (Lippi et al., 2009). These cells release more than 35 cytokines, growth factors, and chemokines that modulate inflammation and promote M2 phenotype microglial activation with neuroprotective properties (Orihuela, McPherson & Harry, 2016), including IL-4 and IL-13 (Davoine & Lacy, 2014). Additionally, eosinophils enhance angiogenesis by secreting vascular endothelial growth factor (Puxeddu et al., 2005). In the present study, inflammatory marker ratios in peripheral blood at admission were assessed for all DFF patients. Several blood-derived inflammatory indices, including NLR and LMR, demonstrated strong associations with the systemic inflammatory response status.

It is worth noting that the Caprini thrombosis assessment model was developed by Dr. Caprini and was first published in 1991. This model is one of the most widely used DVT prediction models in clinical practice. However, according to the Caprini model, the vast majority of DFF patients are classified into the extremely high-risk group, which prevents it from making precise predictions about the occurrence of preoperative DVT. Therefore, this study did not conduct a comparative analysis with the Caprini thrombosis assessment model.

### Clinical value of DVT risk nomogram score model

Currently, most preoperative DVT risk prediction models for patients with lower extremity fractures employ multivariate Logistic regression to identify risk factors, followed by the use of nomograms or risk score equations derived from regression coefficients (Guo et al., 2024). Nomogram models integrate the predictive contribution of each variable and visually present the overall risk estimation, making them widely applicable in clinical prediction research. In this study, a nomogram was developed and validated to enable clinicians to assess preoperative DVT risk in newly admitted DFF patients. The six predictive indicators incorporated into the model were extracted from routinely available clinical data obtained within hours of admission. This tool allows clinicians to rapidly recognize individuals with elevated DVT risk. By aligning each variable’s observed value on its axis and drawing vertical lines to the score scale, the total score and corresponding risk probability can be derived. Patients identified as high-risk should undergo lower extremity color Doppler ultrasonography promptly to confirm DVT, followed by timely pharmacologic intervention to prevent thrombus progression. For patients in the swelling phase, the model supports periodic DVT risk monitoring, enhancing the diagnostic utility of hematological parameters while reducing the frequency and cost of imaging procedures, thereby alleviating financial burden. The strength of this study lies in being the first to identify preoperative lower extremity DVT risk factors in DFF patients based on admission inflammatory markers and to construct a visualized nomogram model. The model demonstrated strong predictive accuracy across multiple performance metrics, with internal and external validation confirming its reliability and clinical applicability.

Several limitations should be acknowledged. The retrospective design and relatively limited sample size may have introduced selection bias. In addition, the single-center nature of the study, with all participants recruited from one institution, restricted the generalizability of the results by failing to account for potential variations across different hospitals or regions. Consequently, the applicability of the conclusions may be uncertain in broader clinical contexts. Future research should involve large-scale, multicenter case-control studies to provide more comprehensive validation, refine the model, and explore the integration of advanced modeling approaches. Furthermore, to reduce subject heterogeneity, patients with multiple or old fractures were excluded during sample selection. Although this improved internal consistency, it also limited the model’s applicability to more complex clinical scenarios.

## Conclusion

This study retrospectively analyzed the preoperative clinical data, hematological inflammatory markers, and their derived ratios in 493 patients with DFF. Independent risk factors for preoperative lower extremity DVT in DFF patients were identified using univariate and multivariate binary logistic regression analyses, and a predictive model was constructed in combination with age. The dataset was randomly divided into training and validation cohorts at a ratio of 7:3. Receiver operating characteristic curves were plotted for both groups, and the area under the curve was calculated for internal validation. The predictive performance of the model was further evaluated using calibration curves and decision curve analysis. In conclusion, NLR, LMR, WBC, NEUT, and LYMPH were significantly associated with an increased risk of preoperative lower extremity DVT in this study. Furthermore, the predictive model based on admission inflammatory markers combined with age showed excellent predictive performance and good reliability, as verified by calibration curves and decision curve analysis.

##  Supplemental Information

10.7717/peerj.21531/supp-1 Supplemental Information 1 Original data

10.7717/peerj.21531/supp-2 Supplemental Information 2 Highlights

## Additional Information and Declarations

### Data Availability

The following information was supplied regarding data availability: The raw data is available in the Supplemental Files.
