# Supplementary material for: Predictive model for preoperative deep vein thrombosis in distal femur fractures using inflammatory blood markers: a single-center retrospective cohort study
Source: PeerJ. 2026 Jul 23;14:e21531. doi: 10.7717/peerj.21531 (PMC13401846; doi:10.7717/peerj.21531)
Supplement: Supplemental Information 2 [file peerj-14-21531-s002.docx]

**Highlights**

· Novel predictive model: We developed and validated the first predictive model for preoperative deep vein thrombosis (DVT) in patients with distal femur fractures (DFF) based on routinely available inflammatory blood indicators.

· Key predictors identified: Neutrophil-to-lymphocyte ratio (NLR), lymphocyte-to-monocyte ratio (LMR), white blood cell count, neutrophil count, lymphocyte count, and eosinophil count were identified as independent determinants of DVT.

· Robust performance: The model demonstrated excellent predictive accuracy, with area under the curve (AUC) values of 0.926 in the training cohort and 0.939 in the validation cohort.

· Clinical utility: The model was rigorously evaluated using calibration curves and decision curve analysis, confirming its strong reliability and potential for clinical application in risk stratification.

· Large cohort: This study is based on a substantial cohort of 493 eligible DFF patients, ensuring the robustness of the findings.
